# Fine ultrastructural features of germ cells and spermatozoa during spermatogenesis in the European grayling, *Thymallus thymallus* (Teleostei, Salmoniformes, Salmonidae)

**DOI:** 10.3389/fvets.2023.1188479

**Published:** 2023-06-01

**Authors:** Hadiseh Dadras, Faranak Dadras, Aiman Imentai, Oleksandr Malinovskyi, Tomáš Pěnka, Jitka Kolářová, Tomáš Policar

**Affiliations:** Faculty of Fisheries and Protection of Waters, South Bohemian Research Center of Aquaculture and Biodiversity of Hydrocenoses, Research Institute of Fish Culture and Hydrobiology, University of South Bohemia in České Budejovice, České Budejovice, Czechia

**Keywords:** electron-dense material, electron microscopy, grayling, spermatogenic cells, ultrastructure

## Abstract

This study aimed to examine the ultrastructure of spermatogenic stages and mature spermatozoa in the European grayling, *Thymallus thymallus*. The testes were examined microscopically with a transmission electron microscope to find out details of the structure and morphology of the grayling germ cells, spermatozoa and some somatic cells. The grayling testis has a tubular shape, with cysts or clusters of germ cells within seminiferous lobules. The spermatogenic cells, including spermatogonia, spermatocytes, and spermatids, can be found along seminiferous tubules. There are electron-dense bodies in germ cells from the primary spermatogonia to secondary spermatocyte stages. These undergo mitosis to reach the secondary spermatogonia stage, when they form primary and secondary spermatocytes. Spermatids undergo three different stages of differentiation during spermiogenesis, characterized by the level of chromatin condensation, elimination of cytoplasm, and the occurrence of the flagellum. The midpiece of spermatozoa is short and contains spherical or ovoid mitochondria. The sperm flagellum has an axoneme with nine doublets of peripheral microtubules and two central microtubules. The result of this study is valuable to be used as a standard reference for germ cell development, which is of great importance to get a clear insight into the process of grayling breeding practice.

## 1. Introduction

The study of germ cell development and segregation and the associated cells, such as Leydig and Sertoli cells, provides valuable information about the reproductive mechanism of spermatogenesis (1–3). The fine structural study of spermatogenesis contributes to a better understanding of germ cell differentiation and elucidates the interactions among different teleost groups, particularly at the family and order levels (4). Furthermore, the study of the ultrastructure of spermatogenic cells during the segregation (pre-meiotic and meiotic) phases of the kinetics of spermatogenesis in teleost fish has indicated they possess certain cytoplasmic features (5, 6).

Testes change morphologically along the annual reproductive cycle of fish, reflecting its seasonality (7, 8). Therefore, studying the ultrastructure of spermatozoa and spermatogenic cells during developmental stages may clarify the reproductive mechanism (2, 9). The ultrastructure of spermatogenesis and the morphology of mature spermatozoa have been examined in many teleost species (10–13) using both light and electron microscopy. However, research on the ultrastructural features of teleost spermatogenesis is scarce; a number of studies have described the ultrastructural details of spermatogenic cells in the common carp, *Cyprinus carpio* (14), silver pomfret, *Pampus argenteus* (14), burbot, *Lota lota* (15), rock flounder, *Kareius bicoloratus* (16), and grey armored catfish, *Liposarcus anisitsi* (17).

European grayling (*Thymallus thymallus*) is a key native freshwater rheophilic fish species in central, northern, and eastern Europe. This species is considered an ecologically valuable bioindicator of water contamination, local habitat, and food supply (18, 19). The European grayling is very popular for sport fishing, and its population has been supported by a long sustainable stocking program, including broodstock management (20) and reproduction (21), and larval and juvenile culture under controlled conditions (22, 23). In this regard, understanding the spermatogenic process and spermatozoa development in the European grayling are important for increasing the knowledge about the reproduction and reproducibility of this species. This knowledge may be useful for the control of programmed maturation of breeder fish and the management of captive breeding and reproduction (24). Because fish fauna is highly diverse, information on the ultrastructural characteristics of spermatogenesis in teleosts is limited to a few species (6, 25). The ultrastructure of grayling spermatozoa has been previously described by Lahnsteiner (22), but the authors did not provide detailed information on the associated cells, such as spermatogenic cells, Sertoli and Leydig cells in this species. Therefore, in this study we have attempted to describe the ultrastructural characteristics of the spermatogenic cell in different stages of development and spermatozoa of the European grayling.

## 2. Materials and methods

### 2.1. Gonad sampling and histology

Eighteen testis samples were collected from a brood stock of three-year-old European grayling males (total length = 336.7 ± 49.3 mm, mass = 255.5 ± 111.8 g). Testes samples were carefully washed with de-ionized water and kept in ice box prior to transfer to the laboratory. Then in the laboratory, the gonads were fixed with Bouin’s solution. After 24 h, the specimens were transferred to 70% ethanol, and the usual histological method was used. Specimens were dehydrated in ascending series of ethanol solutions and then cleaned in a series of xylene solvents (three times). The specimens were then embedded in paraffin and cut into 5 μm sections. The slides containing the samples were stained with hematoxylin and eosin (H&E) and then observed under a light microscope (Leica DM 750, United States).

### 2.2. Transmission electron microscopic observation

To produce tissue specimens for TEM observation of the testicular structure of grayling, the gonads were minced and fixed in 2.5% paraformaldehyde/glutaraldehyde in 0.1 M phosphate buffer solution (pH 7.4) at 4°C for 2 h. Subsequently, the samples were washed thrice with the same buffer solution and postfixed in 2% osmium tetroxide solution in the buffer solution at 4°C for 1 h. The samples were then dehydrated in ascending acetone concentrations (30, 50, 70, 90, 95, and 100%) for 15 min each and embedded in resin (Epon 812, Germany). Ultrathin sections (0.07–0.08 μm) were sliced using a UCT ultramicrotome (Leica Microsystems, Wetzlar, Germany). These were mounted on copper grids, double-stained with uranyl acetate (saturated in 100% alcohol) for 30 min, and counterstained with 1% lead citrate for 20 min. A 1010 transmission electron microscope (JEOL, Tokyo, Japan) operated at 80 kV was used to examine the samples (15, 26).

### 2.3. Ethics statement

This study was conducted in compliance with the Czech Republic’ regulations (law nos. 166/1996 and 246/1992). Further, it was granted permits (nos. 58672/2020-MZE-18134 and 33446/2020-MZE-18134) under the NAZV Project QK22020144. Sampling was carried out with the permission of the Departmental Expert Committee for the Authorization of Experimental Projects of the Ministry of Education, Youth and Sports of the Czech Republic (permit no. MSMT-8155/2022-4).

## 3. Results

### 3.1. Histological observation

**Figure 1:**
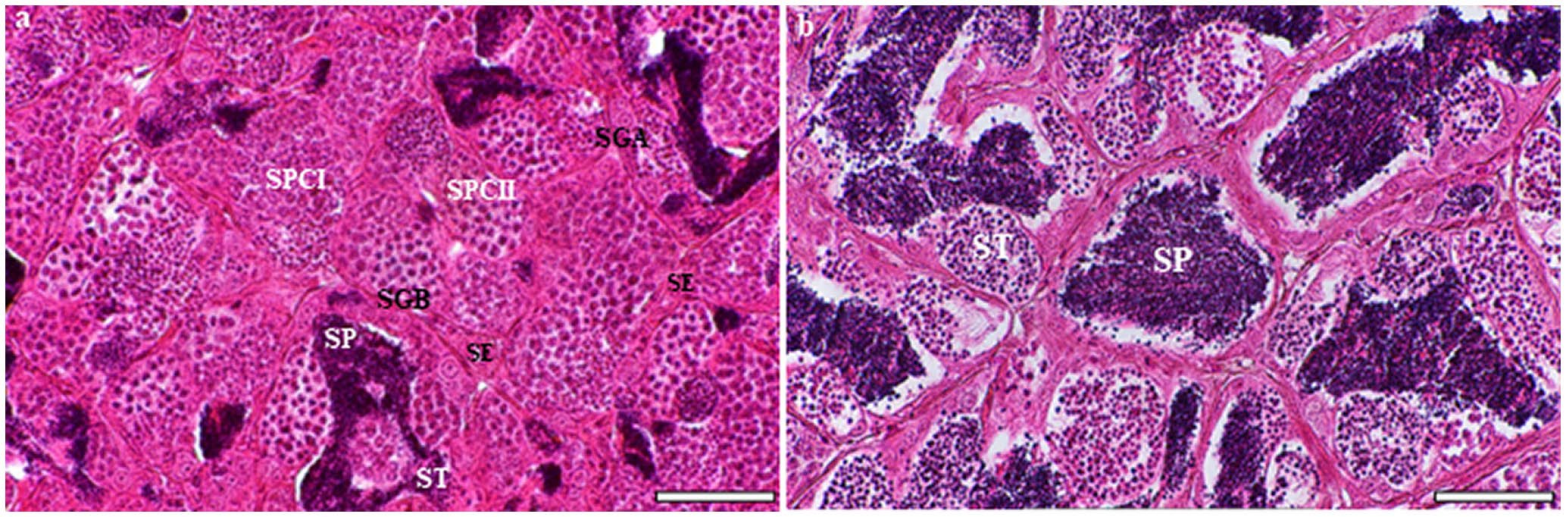
Light microscopic analysis of H&E-stained sections of testes at different developmental stages in the European grayling (*Thymallus thymallus*). **(A)** Transverse section of testicular organization within the seminiferous tubules showing many cysts with different germ cells at multiple sites within these cysts, the location of Sertoli cells and interstitial tissue. **(B)** A higher magnification of tubular lumen filled with spermatids and spermatozoa. SE, sertoli cell; SGA, spermatogonia A; SGB, spermatogonia B; SCI, primary spermatocyte; SCII, secondary spermatocyte, ST, spermatid; SP, spermatozoon. Scale bars: A = 50 μm; B = 30 μm.

Macroscopic examination of grayling testes showed a similar to that of other teleost fishes. Their testes are paired, elongated, and attached to the dorsal wall of the body by a mesorchium. The grayling testis is tubular, with germ cells arranged in clusters inside the seminiferous lobules. Each tubule contains numerous cysts with different germ cells, and spermatogenesis occurs at multiple sites within these cysts. Spermatogonia are in peripherical locations along the length of the lobule, whereas spermatocytes, spermatids, and spermatozoa are found in the interior (Figure 1A). Sertoli cells are located around the germ cells, which they surround with cytoplasmic processes to form cysts; inside these, spermatogenesis occurs. Each tubule is formed by several cysts containing germ cells at different stages of development: spermatogonia A, spermatogonia B, spermatocytes, and spermatids (Figure 1B). While several spermatids and spermatozoa were observed in the tubule lumen, secondary spermatocytes were rarely observed in cysts (Figure 1B).

### 3.2. TEM observation of spermatogenic cells and spermatozoa

TEM observations revealed that, according to their pattern and level of chromatin condensation, germ cells in the spermatogenetic process of the grayling could be classified into ten steps. Primary spermatogonia contain a large spherical nucleus, whereas secondary spermatogonia have small heterochromatin clumps both on the inner surface of the nuclear membrane and in the central area of the nucleus. Primary spermatocytes can be categorized into four subcellular stages, including leptotene, zygotene, pachytene, and diplotene spermatocytes that have thick and long heterochromatin blocks or cords. Secondary spermatocytes contain a nucleus with large blocks of heterochromatin along the inner facet of the nuclear envelope. Spermatids can be divided into three stages.

**Figure 2:**
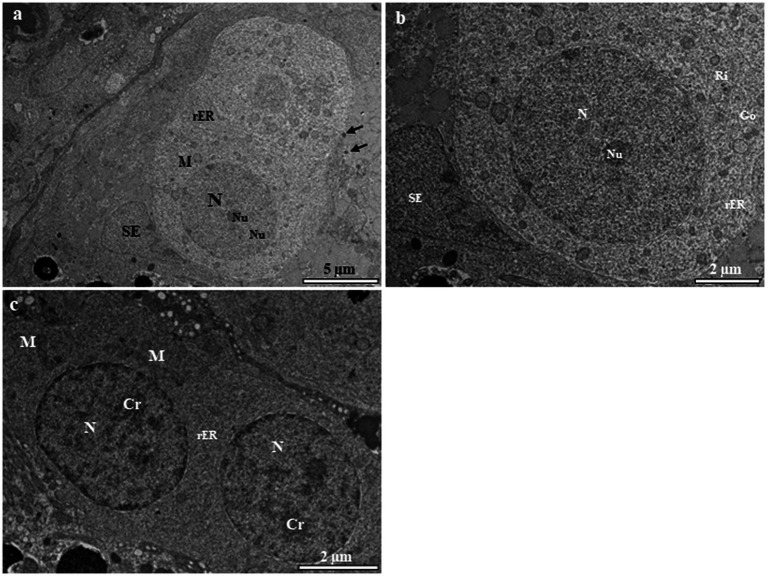
Transmission electron micrographs of spermatogonia in the European grayling. **(A)** Transmission electron micrographs of primary spermatogonia (Sg) showing that the nucleus contains mainly euchromatin surrounded by cytoplasmic processes of Sertoli cells, with a large heterogenic nucleus. The cytoplasm contains few rough endoplasmic reticulum (rER). Circular mitochondria are distributed in the vicinity of the nucleus. Nuage accumulation can be seen close to the nuclear envelope (black arrows). **(B)** Magnified image of primary spermatogonia showing few endoplasmic reticulum, the Golgi complex, and numerous ribosomes and mitochondria in the cytoplasm. **(C)** Transmission electron micrographs of secondary spermatogonia showing small heterochromatin blocks that increase in number and are scattered throughout the nucleus. The cytoplasm contains few endoplasmic reticulum and mitochondria. Cr, chromatin; Go, Golgi complex; M, mitochondria; N, nucleus, Nu, nucleolus; rER, rough endoplasmic reticulum; SE, Sertoli cell.

Primary spermatogonia (Sg1) contain a large spherical nucleus with very few blocks of euchromatin (Figure 2A). Each nucleus contains one or two prominent nucleoli (Figures 2A,B). Most mitochondria are spherical and are found in the cytoplasm, where they tend to congregate in one pole of the cell (Figures 1A,B). Large quantities of the electron-opaque substance termed “nuage” are visible outside the nuclear envelope and float freely in the cytoplasm, either close to the nuclear envelope or in the vicinity of mitochondria (Figure 2A). Secondary spermatogonia (Sg2) are generated by mitotic division of Sg1 and highly resemble their mother cell in an initial stage. Sg2 were identified based on their small size and large amount of dispersed chromatin (Figure 2C). Their nucleus contains more small blocks of heterochromatin than that in Sg1 and these blocks are distributed along the inner facet of the nuclear envelope as well as in the central region (Figure 2C). The mitochondria appear circular or elliptical, with a dense matrix (Figure 2C). The cytoplasm contains fewer mitochondria than that in Sg1, and rough endoplasmic reticulum that is quite evenly distributed in the cytoplasm (Figure 2C).

**Figure 3:**
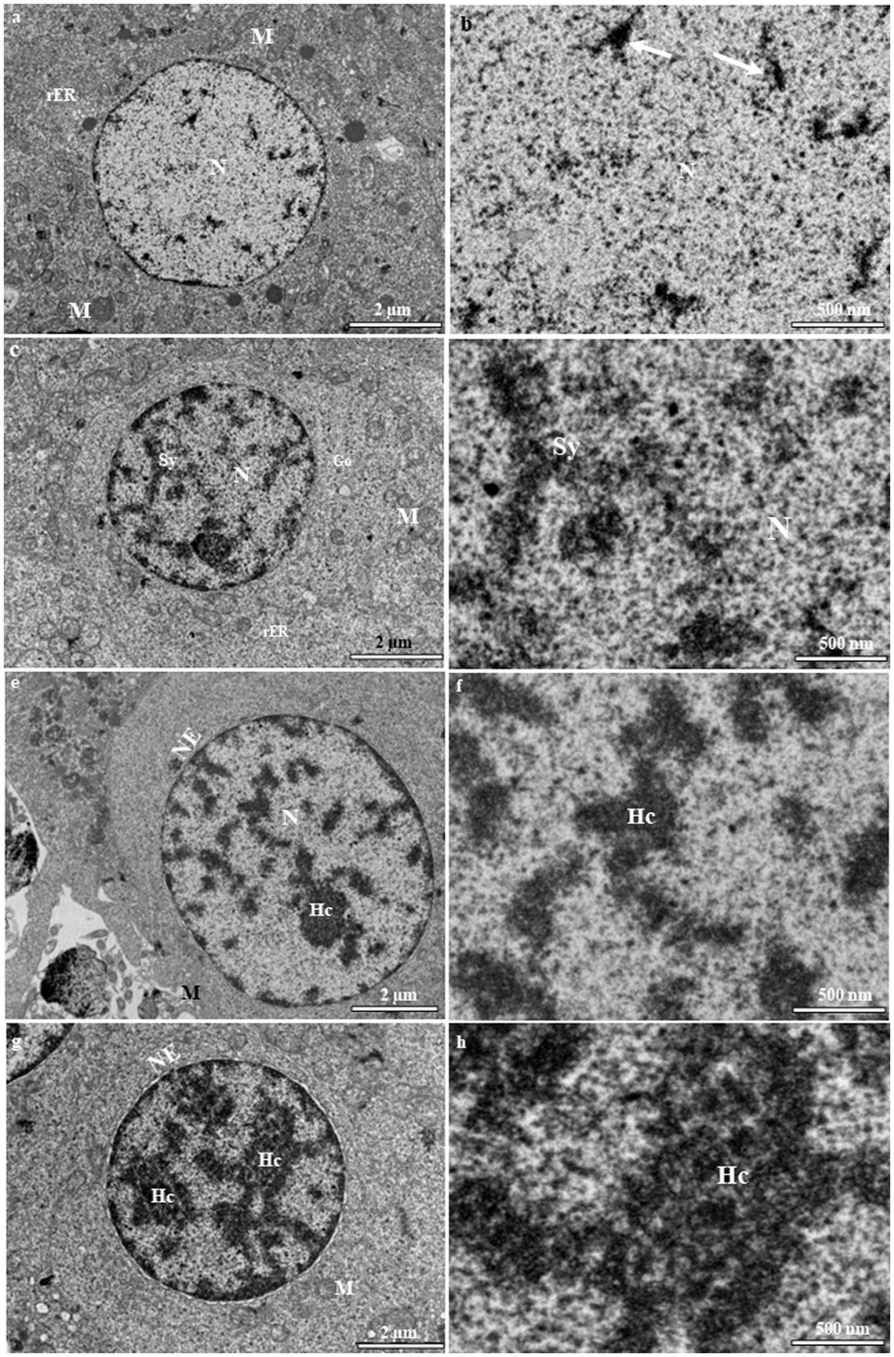
Transmission electron micrographs of primary spermatocyte stages in the European grayling. **(A)** Electron micrograph of leptotene spermatocytes (LSc) with a round nucleus containing mitochondria and rough endoplasmic reticulum. **(B)** Magnified image of leptotene spermatocytes showing that small blocks of condensed chromatin (white arrows) are evenly distributed throughout the nucleus. **(C)** Electron micrograph of zygotene spermatocytes (ZSc) showing that the nucleus contains numerous synaptonemal complexes that are fully formed. The cytoplasm contains mitochondria, the Golgi complex, and rough endoplasmic reticulum. **(D)** Magnified image of the synaptonemal complex in zygotene spermatocytes. **(E)** Electron micrographs of pachytene spermatocytes (PSc) showing the nucleus with long and thick heterochromatin blocks. **(F)** Magnified image of heterochromatin blocks in pachytene spermatocytes. **(G)** Electron micrographs of diplotene spermatocytes (DSc) showing that the nucleus contains long and thick intertwined heterochromatin blocks aligned along the nuclear envelope. **(H)** Magnified image of heterochromatin blocks in diplotene spermatocytes. Hc, heterochromatin; M, mitochondria; N, nucleus; NE, nuclear envelope; F, flagellum; M, mitochondrion; N, nucleolus; rER, rough endoplasmic reticulum; Sy, synaptonemal complex.

**Figure 4:**
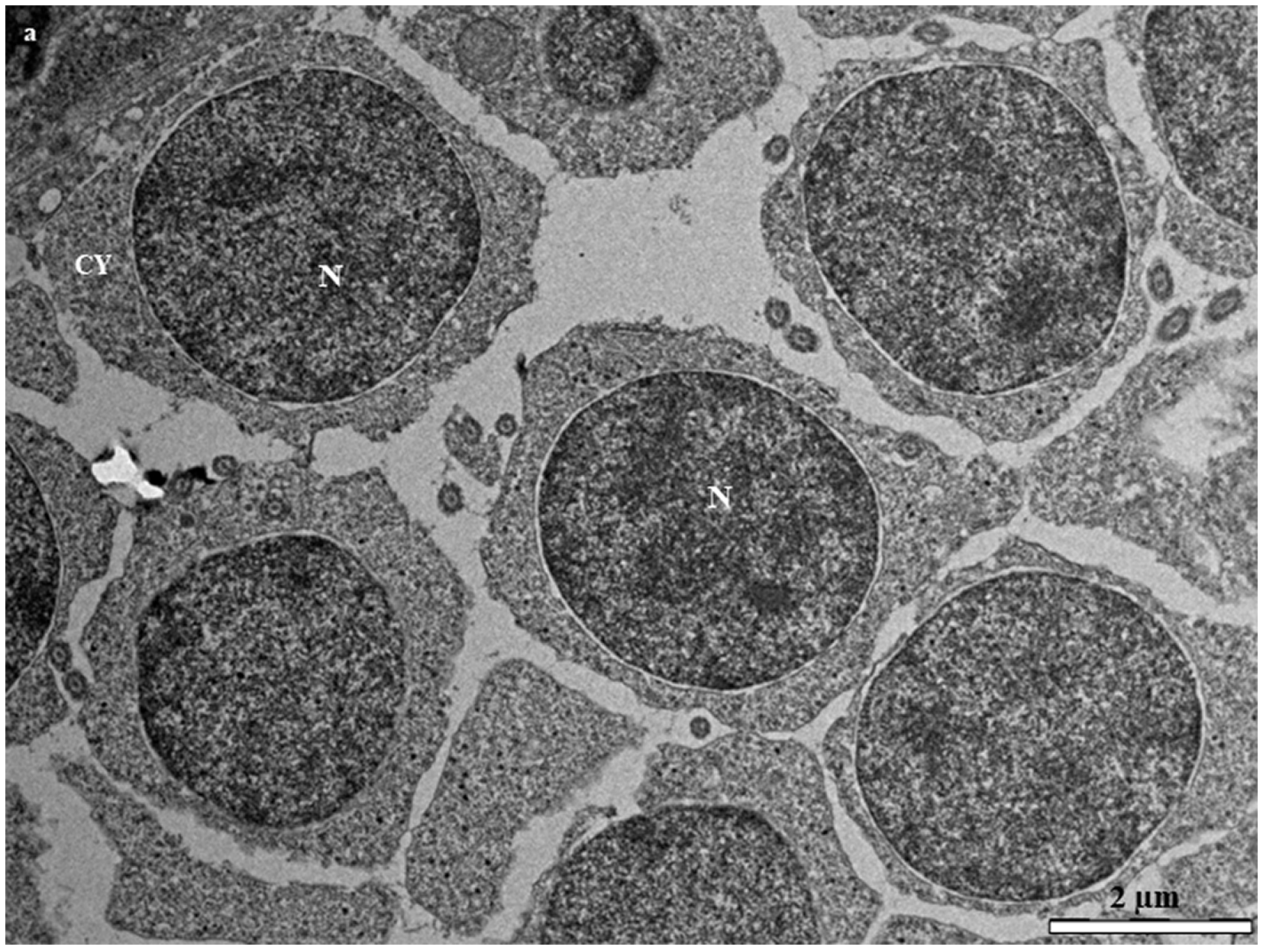
Transmission electron micrographs of secondary spermatocytes in the European grayling showing a large electron dense heterogenic nucleus and high level of chromatin condensation scattered throughout the nucleus. CY, cytoplasm; M, mitochondria; N, nucleus.

Primary spermatocytes are categorized into leptotene (LSc), zygotene (ZSc), pachytene (PSc) and diplotene (DSc) spermatocytes based on their distinct patterns of chromatin organization. The early LSc is spherical in shape, with a large spherical nucleus (Figure 3A). Small loosely packed chromatin blocks are distributed evenly in the euchromatic area (Figure 3A). In the late LSc, the chromatin blocks in the nucleus become larger, indicating the beginning of chromatin condensation (Figure 3B). The ZSc has a round nucleus of roughly the same size as that in the LSc. The distinctive feature of ZSc is the increased size and density of the heterochromatin blocks, which are distributed along the synaptonemal complex (Figures 3C,D). The nucleolus has completely disappeared in the ZSc (Figure 3D). The distinctive features of the PSc are the round nucleus and the existence of long interconnecting cords of heterochromatin, some of which connect to the nuclear envelope at their ends (Figure 3E). Some of these cords remain linked to the synaptonemal complex (Figure 3E). PSc are the most numerous cell type in the seminiferous tubules and are easy to observe owing to their unique characteristics mentioned above. The cytoplasm also contains ribosomes, mitochondria, and rough endoplasmic reticulum. The nucleus of the DSc cell is nearly round to oval. The chromatin blocks become increasingly larger and are connected to the nuclear envelope (Figure 3F). DSc cells are considerably less abundant than PSc cells; however, the types of cytoplasmic organelles appear similar to those observed in PSc cells. The first meiotic division of a primary spermatocyte produces two secondary spermatocytes, which are not usually observed in histological sections, indicating that the duration of a spermatogenic cell in the secondary spermatocyte stage is short-lived (Figures 3G,H). The secondary spermatocyte is smaller than the primary spermatocyte, with a reduced cytoplasm. The nucleus of a secondary spermatocyte is round, while the nucleolus is not evident, and heterochromatin material (chromatin) is frequently observed in the nucleus (Figure 4).

**Figure 5:**
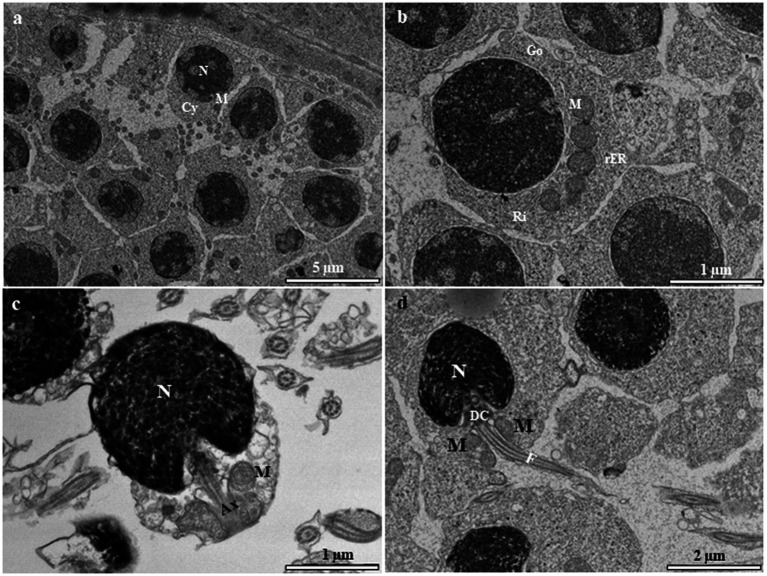
Transmission electron micrographs of spermatid stages in the European grayling. **(A)** Transmission electron micrograph of newly formed spermatids (STI) showing an irregularly distributed electron-dense nucleus as fine granules. **(B)** Magnified image of spermatid I (St1) showing cytoplasm that contains mitochondria, relatively few ribosomes, rough endoplasmic reticulum, and a few stacks of Golgi complex. **(C)** Transmission electron micrograph of spermatid II (St2) exhibiting an oval-shaped nucleus, while the developing tail appears as an axoneme growing out from the basal body at the posterior end. **(D)** The final stage of spermatid differentiation showing the developing flagellum connected to the distal centriole and mitochondria in the vicinity of the midpiece. Ax, axenome; CY, cytoplasm; DC, distal centriole; F, flagellum; M, mitochondria; N, nucleus; CY, cytoplasmic; M, mitochondrion, N, nucleous; Ri, ribosomes; Rer, rough endoplasmic reticulum.

There are three stages in spermatid differentiation during spermiogenesis, i.e., spermatid I (St1), spermatid II (St2), and spermatid III (St3), which are classified according to the elimination of organelles and cytoplasm, flagellum formation, patterns of chromatin condensation, and the occurrence of the flagellum. The nuclei of successive spermatid stages vary from round to oval and finally, cylindrical. St1 are grouped in cysts that lie close to the lumen (Figure 5A). Each cell is characterized by the presence of a round to oval nucleus (Figure 5A). The cytoplasm is a relatively large mass, and the nucleus is reduced in size, with abundant mitochondria that are gathered in the vicinity of the nucleus (Figure 5B). St2 are smaller than St1, with more compact chromatin in the nucleus. The nucleus of St2 is decreased in size, oval-shaped, and eccentrically located within the cell (Figure 5C). Globular-shaped mitochondria with tubular cristae are widely dispersed throughout the cytoplasm (Figure 5C). In St3, the nucleus is elongated and is shaped as that in spermatozoa, containing completely condensed chromatin (Figure 5D). Circular or oval mitochondria with an electron-dense matrix lie close to the nucleus (Figure 5D). The excess spermatid cytoplasm is eliminated (Figure 5D).

**Figure 6:**
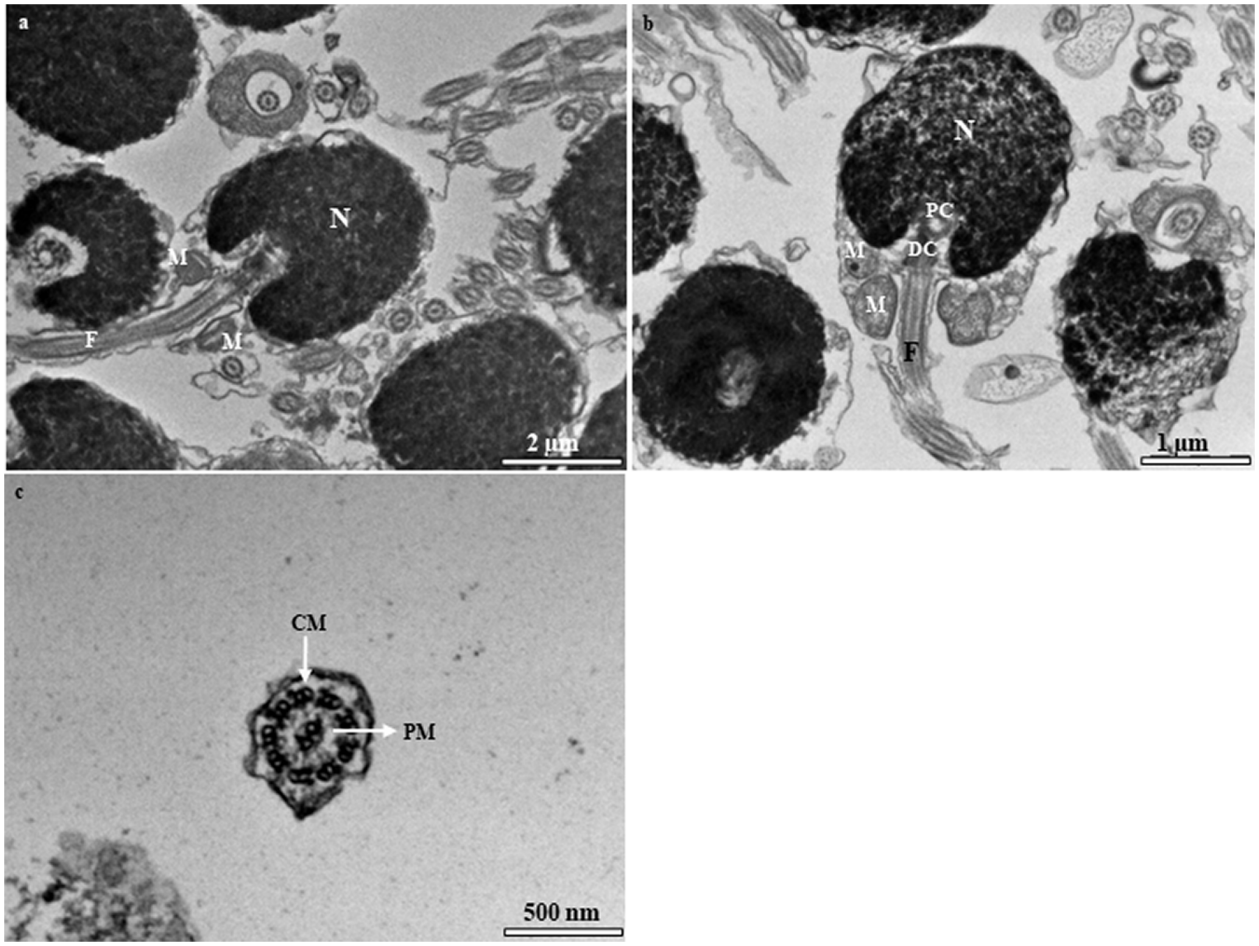
Transmission electron micrographs of fresh spermatozoa in the European grayling. **(A)** Transmission electron micrograph of mature spermatozoa displaying different parts of the cell. **(B)** Magnified image of a spermatozoon and midpiece showing the distal centriole (DC) and proximal centriole and mitochondria. **(C)** Magnified image of the axoneme of the flagellum showing the typical structure of 9 peripheral microtubules (PM) + 2 central microtubules CM of the axonemal doublet. CM, central microtubule; F, flagellum; M, mitochondria; N, nucleolus; PC, proximal centriole; PM, peripheral microtubular distal centriole.

Mature spermatozoa (SZ) are characterized by their elongated oval nuclei composed of a head, short midpiece, and single flagellum (Figure 6A). The mature spermatozoon is oval in shape, with condensed electron-dense heterochromatin material in the nucleus, and there appear two centrioles beneath the nucleus (Figure 6A). The midpiece is short and contains spherical and ovoid mitochondria. The flagellum is enveloped by the flagellar plasma membrane that extends into the cytoplasm in cytoplasmic canals around the midpiece (Figure 6B). The axoneme contains 8–10 mitochondria surrounding the flagellar axial filament and is observed around the midpiece of spermatozoon (Figure 6C).

**Figure 7:**
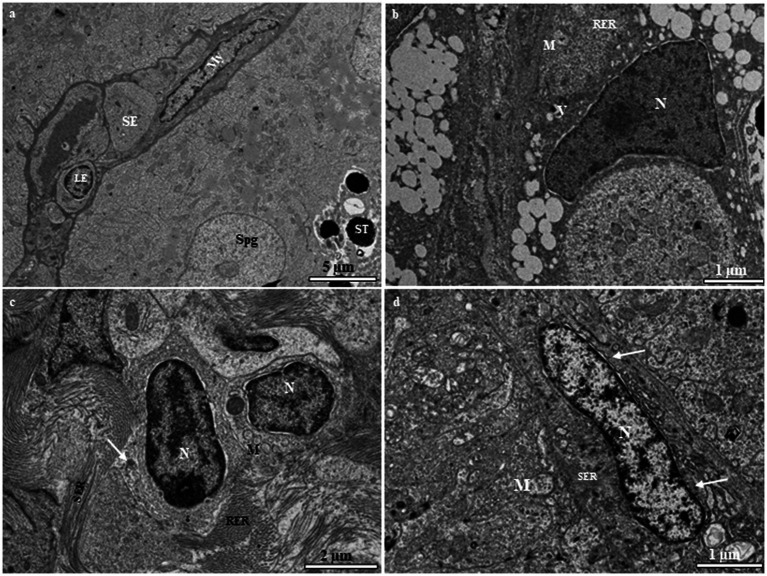
**(A)** Transmission electron micrograph of the European grayling testis showing Sertoli cells surrounding spermatogonia and spermatids and other cystic cells, including Leydig and myoid cells, separated from the germinal compartment by the basement membrane. **(B)** Magnified image of a Sertoli cell showing the large triangular nucleus, rough endoplasmic reticulum, mitochondria, and vacuole. **(C)** Magnified image of a Leydig cell showing a large irregular nucleus with electron-dense chromatin, mitochondria, and variable-sized lipid droplets (white arrow). **(D)** Magnified image of a myoid cell with numerous microfilaments (white arrows) surrounding the heterochromatin-rich nucleus. CY, cytoplasm; ESR, smooth endoplasmic reticulum; LY, Leydig cell; M, mitochondria; My, myoid cell; N, nucleus; SE, Sertoli cell; Spg, spermatogonia; ST, spermatid; V, vacuole.

Sertoli and Leydig cells, which are somatic cells, are observed within the tubules. Sertoli cells surround the spermatogonia and spermatid cells (Figure 7A). The nucleus of the Sertoli cell appears roughly triangular and is located near the basal lamina (Figure 7A). The cytoplasm contains rough and smooth endoplasmic reticulum cisternae, typical mitochondria, and a number of vesicular structures that are not attached to the endoplasmic reticulum (Figure 7B). The interstitial tissue is involved in the formation of the outside scaffold for the testis tubules and contains constituents of connective tissues, including fibrocytes, collagenous fibers, Leydig cells, myoid boundary cells, and blood vessels (Figure 7A). The Leydig cell contains a large irregular nucleus with clumps of electron-dense material and electron-lucent cytoplasm in which the smooth endoplasmic reticulum is scattered with mitochondria (Figure 7C). The myoid cells are typically located near the basal lamina (Figure 7D). The nucleus of these cells is usually elongated and heterochromatin-rich (Figure 7D). The cytoplasm contains small mitochondria and numerous microfilaments surrounding the heterochromatin-rich nucleus (Figure 7D).

## 4. Discussion

Cystic spermatogenesis is a characteristic feature of most teleosts. The present study revealed that the type of spermatogenesis in the grayling is cystic spermatogenesis, as described by Billard (27). Each cyst in the lobular lumen contains germ cells in different developmental stages, as observed in grey armored catfish, *Liposarcus anisitsi* (17) and burbot, *Lota lota* (15). According to Parenti and Grier (28), this pattern of spermatogenic development is known as a restricted-type testicular structure in which spermatogonia are restricted to the tube walls (28–30), and the gonad can be classified as a tubular anastomosing type with an unrestricted distribution of spermatogonia. This type of gonadal structure has also been identified in the northern pike, *Esox Lucius* (29, 31), catfish, *Conorhynchus confronters* (32), zebrafish, *Danio rerio* (9), and burbot (15). The presence of germ cells within cysts is a pattern of spermatogenic development that occurs in most teleosts. In this study, synchronous differentiation of germ cells was observed during spermatogenesis in the grayling, and it was confirmed that most stages of spermiogenesis proceed within cysts. The only sperm cells that are released into the tubule lumen are those that have undergone complete differentiation. Interestingly, this pattern of germ cell development is frequently observed in teleost testes.

As in most teleost fish, spermatogenesis in the grayling starts with Sg1. Sg1 are considered the largest germ cells, with electron-lucent cytoplasm and a large round to oval nucleus with a prominent nucleolus. During cell division, a several morphological changes occur leading to the transformation of Sg1 into sperm, with increasing cell numbers and decreasing cell size. According to our observation, particularly in earlier stages of cell development, an electron-dense substance appears in the cytoplasm near the nuclear envelope of the Sg1 as seen in other teleost species, such as the piper gurnard, *Trigla lyra* (33), stone flounder, *Kareius bicoloratus* (16), silver pomfret, *Pampus argenteus* (34) and burbot (15). In this study, nuage was observed in Sg1 and secondary spermatocytes. Electron microscopic observation of nuage has been reported in the cytoplasm of the secondary spermatocytes of Lake Magadi tilapia, *Alcolapia grahami* (35) and the great blue spotted mudskipper, *Boleophthalmus pectinirostris* (36). However, its function has not been uncovered. The most distinctive feature of the primary spermatocytes was the presence of synaptonemal complexes, which are protein structures, in the nucleus; they are thought to take part in the homologous chromosome pairing step in meiosis (37, 38). This characteristic of primary spermatocytes is also observed in *Trahira Hoplias malabaricus* (39), the Manila clam, *Ruditapes philippinarum* (40), and *Acrossocheilus fasciatus* (10).

Spermiogenesis is characterized by preparatory morphological changes and modifications of the spermatids, including structural changes (41–43). The present study showed that the process of spermatid development and their morphology in the grayling involves the elimination of organelles and cytoplasm, flagellum formation, patterns of chromatin condensation, and the occurrence of the flagellum, as reported in many teleosts (12, 15, 44, 45). During spermatid development, the chromatin condensation matures in a definite pattern, always beginning close to the developing flagellum. According to Quagio-Grassiotto and Oliveira (42), three types of spermiogenesis in fish (types I, II, and III) have been demonstrated based on the orientation of the flagellum to the nucleus and on whether or not a nuclear rotation occurs. These patterns in the spermatozoal structure of teleosts are fixed, highly conserved within taxonomic units, and considered a valuable tool for phylogenetic analyses in fish (42, 46). According to our results, grayling spermatids are of type II, showing flagellum development parallel to the nucleus without nuclear rotation, which has been reported in several fish species (12, 47–49). The final stage of spermiogenesis is the formation of mature spermatozoa. The head region of the teleostean spermatozoon consists of a nucleus containing condensed chromatin. Spermatozoa differ in form depending on the species (50–52). In the grayling, the head region of the spermatozoon consists of an oval nucleus with condensed chromatin, which has been reported in other species, such *Stanoperca* sp. Mullidae and Siganidae (53), the blue spart, Clupeidae (54), and paradise fish, *Macropodus opercularis* (44). As in other teleosts, the grayling spermatozoon flagellum has a typical 9 + 2 microtubular structure.

Our observations showed that Sertoli cells exist on the borders of the cysts that contain the Sg1, Sg2, and spermatids. The Sertoli cell is triangular, with a large electron-dense nucleus. The second type of somatic interstitial cells, known as Leydig cells, are formed between the seminiferous tubules. They have a large irregular nucleus with clumps of electron-dense material, some of which are located on the nuclear membrane. They have electron-lucent cytoplasm in which smooth endoplasmic reticulum, mitochondria, and free ribosomes are scattered. This observation of Leydig cells at the ultrastructural level has been described in many fish species (7, 15, 34, 36). The third type of somatic cells are myoid cells, which have ultrastructural features similar to those in the cichlid, northern pike, and common carp (4, 55, 56). The detailed information in this report illustrates the importance of germ cell development in this species, which can be used to improve the performance of grayling breeding practice.

## Data availability statement

The original contributions presented in the study are included in the article/supplementary material, further inquiries can be directed to the corresponding author.

## Ethics statement

The animal study was reviewed and approved by this study was conducted in compliance with the valid legislative regulations of the Czech Republic (law nos. 166/1996 and 246/1992), and was granted permits (nos. 58672/2020-MZE-18134 and 33446/2020-MZE-18134 under the NAZV Project QK22020144). All samples were collected with the permission of the Departmental Expert Committee for the Authorization of Experimental Projects of the Ministry of Education, Youth and Sports of the Czech Republic (permit no. MSMT-8155/2022-4).

## Author contributions

HD: conceptualization, investigation, and writing – original draft preparation. HD and AI: methodology. HD, AI, OM, TPe, JK, and TPo: sample collection. HD, FD, and AI: formal analysis and data curation. HD: re-sources. HD, FD, and TPe: writing – review and editing. TPo: project administration and supervision. All authors contributed to the article and approved the submitted version.

## Funding

This research was funded by the Ministry of Agriculture of the Czech Republic, Project NAZV QK22020144.

## Conflict of interest

The authors declare that the research was conducted in the absence of any commercial or financial relationships that could be construed as a potential conflict of interest.

## Publisher’s note

All claims expressed in this article are solely those of the authors and do not necessarily represent those of their affiliated organizations, or those of the publisher, the editors and the reviewers. Any product that may be evaluated in this article, or claim that may be made by its manufacturer, is not guaranteed or endorsed by the publisher.
